# The Navicular Graft for Reconstructing Deep Alar Defects

**DOI:** 10.7759/cureus.16657

**Published:** 2021-07-27

**Authors:** Caitlin Robinson, Nader Aboul-Fettouh, Timothy Hansen, Sogyong L Auh, Deborah F MacFarlane

**Affiliations:** 1 Dermatology, Colorado Springs Dermatology Clinic, Colorado Springs, USA; 2 Dermatology, University of Texas Health Science Center at Houston, Houston, USA; 3 Dermatology, McFarland Clinic, Ames, USA; 4 Dermatology, Premier Dermatology, Crest Hill, USA; 5 Dermatology, MD Anderson Cancer Center, Houston, USA

**Keywords:** reconstruction, mohs surgery, nasal ala, full-thickness skin graft, skin graft, skin cancer

## Abstract

The reconstruction of deep nasal ala defects can be challenging. The often thick, sebaceous skin of the nose provides structural support helping maintain the ala shape and nasal patency; loss of this support may result in ala deformity and nasal vestibule collapse. Traditional full-thickness skin grafts of deep alar defects may result in depressed scars. We present a variation of the full-thickness skin graft to repair deeper alar defects, sculpting the graft into a boat-shaped or “navicular” form. This allows for sufficient volume restoration and good cosmesis while avoiding more extensive surgical repairs of the nasal ala. The navicular graft offers several advantages: the avoidance of more extensive procedures involving cartilage grafts and/or flaps, appropriate color/texture match, and volume restoration without pitting, notching, or retraction of nasal structures. In addition, no struts or bolsters are needed.

## Introduction

Reconstruction of deep nasal ala defects can present a challenge. The often thick, sebaceous skin of the nose provides structural support helping maintain ala shape and nasal patency; loss of this support may result in ala deformity and nasal vestibule collapse. The granulation repair of deep alar defects may result in alar retraction and poor cosmesis, while local flaps may sometimes cross cosmetic subunits and result in trapdoor deformity or alar rim distortion [1]. Traditional full-thickness skin grafts (FTSGs) of deep alar defects may result in depressed scars [2].

While traditional teaching endorses defatting FTSGs, several studies have demonstrated that the inclusion of subcutaneous fat may reliably restore contour without significantly affecting graft survival [3,4]. We introduce the concept of sculpting the graft into a boat-shaped or “navicular” form, which allows for sufficient volume restoration and good cosmesis while avoiding more extensive surgical repairs of the nasal ala.

## Case presentation

**Figure 1:**
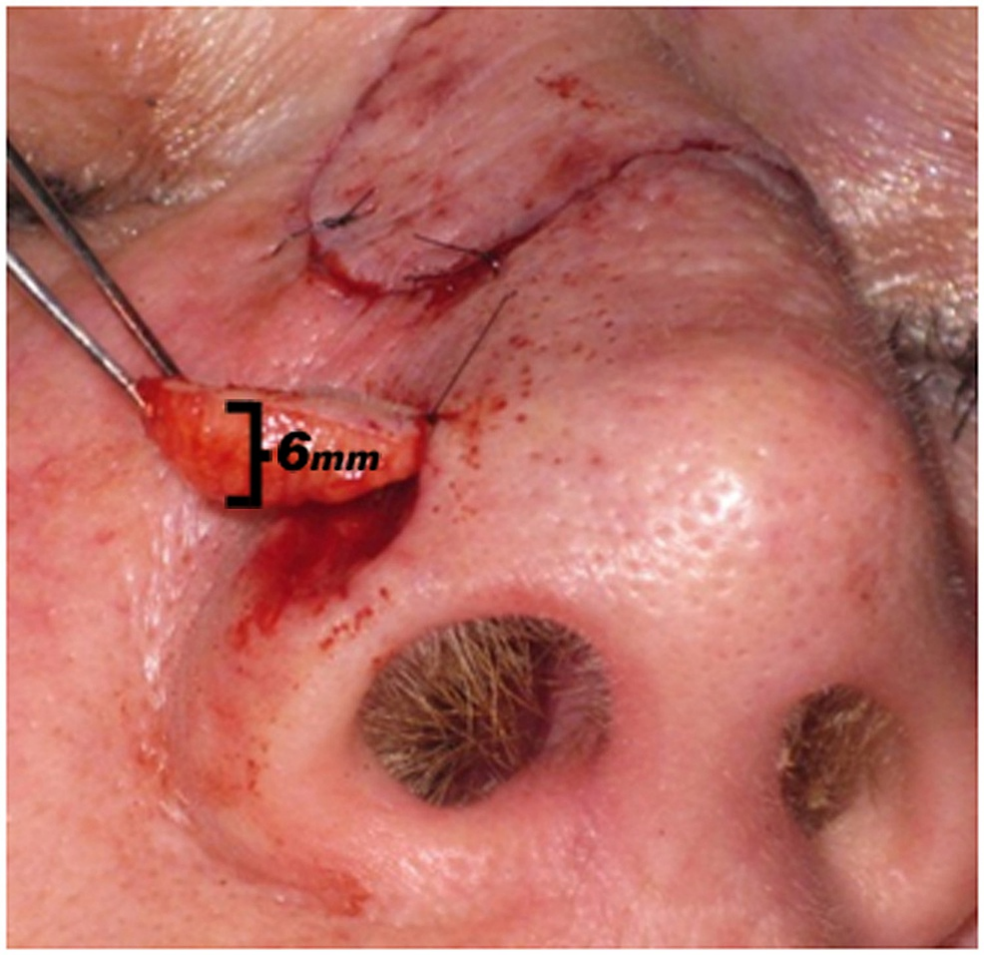
A full-thickness skin graft harvested from the glabellar skin The graft was sculpted into a “boat” shape, which filled the depth of the defect and was slightly narrower than the defect diameter.

Three patients, aged 63 to 78 years, underwent Mohs micrographic surgery on the nasal ala with deep defects that required closure. FTSGs were harvested from the nasolabial fold in two patients and glabellar skin (from a concurrent repair) in one patient. The grafts were sculpted into a boat-like shape, which filled the depth of the defect and were slightly narrower than the defect diameter (Figure 1). Rather than thinning the graft, sufficient subcutaneous tissue was left to replace the defect volume. Each graft was individually sculpted to match the depth of the defect and contour of the surrounding skin. The grafts were sutured in place with interrupted and continuous 6-0 nylon sutures, and dressings were placed without additional bolstering. Patients' postoperative results were documented periodically for up to eight months. 

**Figure 2:**
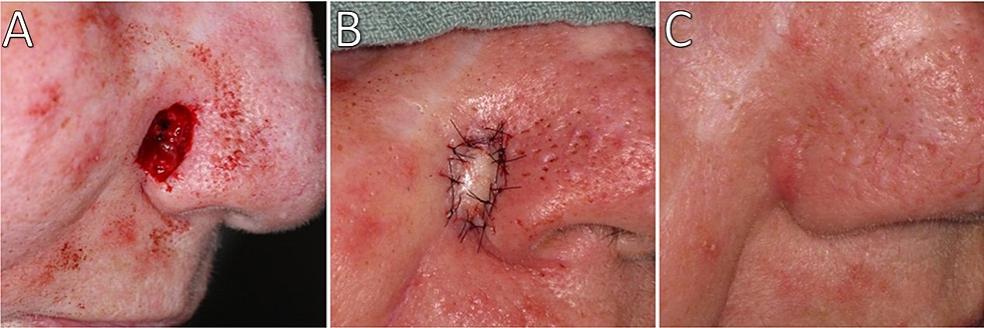
Patient 1 (A) Post-surgical defect. (B) Navicular graft sutured in place. (C) At four-month follow-up.

**Figure 3:**
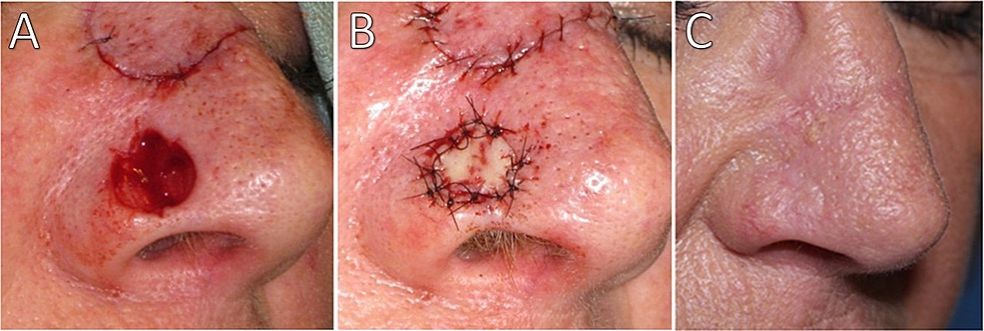
Patient 2 (A) Post-surgical defect. (B) Navicular graft sutured in place. (C) At seven-month follow-up.

**Figure 4:**
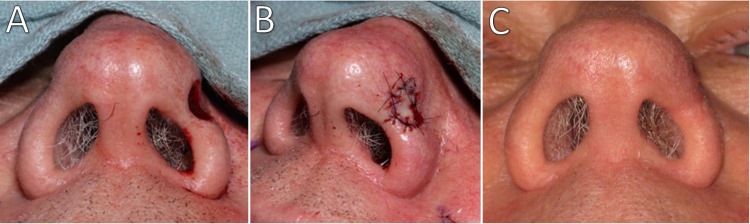
Patient 3 (A) Post-surgical defect. (B) Navicular graft sutured in place. (C) At one-month follow-up.

Surgical defects measured 8-12 mm in diameter and 4-7 mm in depth (Table 1). The follow-up examination revealed well-healed grafts with good restoration of the nasal contour and no notching or impairment of alar patency (Figures 2-4). No scar revision procedures were required, and all patients were very satisfied with the outcomes.

**Table 1:** Surgical defect size and navicular graft donor site details

Patient	Age (years), gender	Defect size (length x width x depth)	Donor site
Patient 1	76, female	1.2 x 1.0 x 0.7 cm	Nasolabial fold
Patient 2	63, female	1.2 x 1.2 x 0.6 cm	Glabella
Patient 3	78, male	1.0 x 0.8 x 0.4 cm	Nasolabial fold

## Discussion

Deep defects of the nasal alae present reconstructive challenges due to complex anatomic topography, nearby free margins, and minimal elasticity of nearby thick sebaceous skin. The use of traditional full-thickness skin grafts for lower nose reconstruction has been described, but recommendations are typically limited to shallow and small defects <1 cm [3].

Many authors argue that optimizing graft survival requires fully defatting the graft to allow for adequate imbibition and inosculation [4]. This can result in overly thinned grafts, which may be hypopigmented and depressed relative to surrounding skin edges. Several publications have shown that the inclusion of subcutaneous fat with FTSGs, when performed in the right context, can reliably restore contour without significantly affecting graft survival [1-4]. Our findings presented herein further substantiate this claim.

With careful selection of the donor site, color and texture match can be optimized, but challenges remain with restoring contour. Our experience supports the practice of sculpting each graft to precisely fit the contour of the defect, trimming subcutaneous tissue only as necessary to position the graft flush to the surrounding skin.

It has been previously reported by Shimizu and MacFarlane that tie-over bolsters, though traditionally recommended to increase skin graft survival by providing hemostasis and immobilization, may not be necessary to ensure the survival of FTSGs, and this observation is further supported herein [5]. Within skin-fat composite grafts, bolsters may even suppress edema that is necessary for grafts’ survival and proper neovascularization [2]. Bolsters can also be cumbersome for the patient.

## Conclusions

We present a variation of the full-thickness skin graft to repair deeper alar defects. We have used the term “navicular” due to the boat-like shape of the graft. This method combines the principle of a composite (skin-fat) graft and post-harvest sculpting to maximize aesthetic outcomes while potentially avoiding more extensive procedures. Nasolabial or glabellar skin is ideally used due to its sebaceous nature and adequate thickness, allowing the sculpting necessary to match the defect depth. The navicular graft offers several advantages: the avoidance of more extensive procedures involving cartilage grafts and/or flaps, appropriate color/texture match, and volume restoration without pitting, notching, or retraction of nasal structures. In addition, no struts or bolsters are needed.
